# Long-Chain Hydrocarbons (C21, C24, and C31) Released by *Bacillus* sp. MH778713 Break Dormancy of Mesquite Seeds Subjected to Chromium Stress

**DOI:** 10.3389/fmicb.2020.00741

**Published:** 2020-04-24

**Authors:** Verónica Ramírez, José-Antonio Munive, Luis Cortes, Jesús Muñoz-Rojas, Roberto Portillo, Antonino Baez

**Affiliations:** ^1^Centro de Investigaciones en Ciencias Microbiológicas, Instituto de Ciencias, Benemérita Universidad Autónoma de Puebla, Puebla, Mexico; ^2^Facultad de Ciencias Químicas, Benemérita Universidad Autónoma de Puebla, Puebla, Mexico

**Keywords:** bacterial volatile, seed dormancy, chromium stress, growth promotion, *Prosopis laevigata*

## Abstract

Volatile organic compounds (VOCs) produced by rhizobacteria have been proven to stimulate plant growth during germination and seedling stages. However, the modulating effect of bacterial volatiles on the germination of seeds subjected to heavy metal stress is scarcely studied. In this work, the ability of volatiles released by *Bacillus* sp. MH778713 to induce seed dormancy breakage in *Prosopis laevigata* and *Arabidopsis thaliana* seeds were examined. The minimal inhibitory concentration of chromium (Cr) VI that prevents seed germination of *P. laevigata* and *A. thaliana* on water-Cr-agar plates was 2500 and 100 mg L^–1^, respectively. Remarkably, partitioned Petri-dish co-cultivation of *Bacillus* sp. MH778713 and plant seeds under Cr-stress showed the beneficial effect of volatiles emitted by *Bacillus* sp. MH778713, helping plant seeds to overcome Cr-stress. Among the metabolites emitted by *Bacillus* sp. MH778713, octadecane, heneicosane, 2,4-di-tert-butylphenol, hexadecane, eicosane, octacosane, and tetratriacontane were the most abundant. To confirm that these long-chain compounds produced by *Bacillus* sp. MH778713 could be responsible for the seed dormancy breakage, high pure organic compounds (2,4-di-tert-butylphenol, heneicosane, hentriacontane, and tetracosane) were used directly in germination assays of *P. laevigata* and *A. thaliana* seeds instead of volatiles emitted by *Bacillus* sp. MH778713. All organic compounds allowed *Prosopis* and *Arabidopsis* seeds to overcome Cr-toxicity and germinate. The results of this study provide new insight into the role of long-chain bacterial compounds produced by *Bacillus* sp. MH778713 as triggers of seed abiotic stress tolerance, surmounting chromium stress and stimulating seedling development.

## Introduction

Plant growth-promoting rhizobacteria (PGPR) are beneficial plant symbionts capable of providing plants with improved water and nutrient uptake (Vacheron et al., 2013; Backer et al., 2018). Rhizobacteria are intimately associated with the root system of the plants and can affect the growth and fitness of plants by modulating nutrient uptake, inducing systemic resistance, and tolerance to abiotic and biotic stress (Bitas et al., 2013; Backer et al., 2018). PGPR can produce auxins, cytokinins, and gibberellins that regulate plant growth and development. Furthermore, each rhizobacterial species produce a specific blend of volatile organic compounds (VOCs) with important roles in plant biology and bacterial life cycle (Ryu et al., 2003; Sharifi and Ryu, 2018).

Bacterial volatiles can modulate root architecture, iron uptake, sodium and auxin homeostasis in plants (Zhang et al., 2007; Gutierrez-Luna et al., 2010; Liu and Zhang, 2015). Thus, salt-stressed plants treated with *Bacillus amyloliquefaciens* VOCs show greater Na^+^-stress tolerance than control plants (Zhang et al., 2008). Similarly, drought-stressed plants exposed to *B. subtilis* GB03 VOCs were more tolerant than plants without VOCs treatment due to the accumulation of osmoprotectants such as choline and glycine (Zhang et al., 2010; Liu and Zhang, 2015). The volatile blend release by *Bacillus subtilis* GB03 can also induce systemic resistance in *Arabidopsis* against *Erwinia carotovora* (Ryu et al., 2004). To date, the demonstrated efficacy of VOCs in enhancing plant fitness and tolerance to salinity, drought, iron deficiency, and sulfur starvation stress suggest that bacterial volatiles might help plants to tolerate other abiotic stresses, but their effectiveness should be confirmed for each stress condition.

Seed dormancy and germination are complex processes regulated mainly by the plant hormones gibberellins (GAs) and abscisic acid (ABA). Environmental and endogenous factors might control the abundance of signaling molecules which determine the ABA/GAs ratio, thus elevated ABA concentration within seeds triggers seed dormancy while high GAs level induces germination (Bailly et al., 2008; Graeber et al., 2012; Li et al., 2018). There are different definitions and classifications of seed dormancy; a delay or fail to germinate under favorable conditions is a generally accepted definition (Arc et al., 2013). Several abiotic stresses like salinity, drought, cold, and exposure to heavy metal can induce ABA biosynthesis and seed dormancy (Vishal and Kumar, 2018). Indeed, arsenic and vanadium up-regulate ABA biosynthesis genes of rice roots while cadmium increases endogenous ABA levels (Bücker-Neto et al., 2017). Germination of wheat, cucumber, and chickpea seeds decreases by exposure to copper (Cu^2+^), zinc (Zn^2+^) or lead (Pb) whereas abscisic acid content increase; suggesting that ABA plays an important role not only on seed germination but also in stress response (Munzuroğlu et al., 2008; Wang et al., 2014; Bücker-Neto et al., 2017).

Chromium (Cr) is a non-essential and toxic element to plants and animals. The most ubiquitous oxidation states of Cr in nature are trivalent (Cr III) and hexavalent (Cr VI) chromium; being Cr(VI) the most phytotoxic (Panda and Choudhury, 2005). Chromium(VI) at concentrations ranged 50–100 mg kg^–1^ soil are toxic for most plants affecting germination rate, chlorophyll content, and most of the growth parameters (Akinci and Akinci, 2010; Jain et al., 2016; Amin et al., 2019). For plants growing in a nutrient solution, 10–25 mg L^–1^ Cr VI is detrimental for plant development (Dube et al., 2003; Ramírez et al., 2019). Nonetheless, *Prosopis* plants can tolerate, translocate, and hyper accumulate heavy metals, including Pb, As and Cr^6+^ (Aldrich et al., 2003, 2007; Jayaram and Prasad, 2009). We reported that *Prosopis* trees grown in polluted wildlife habitats are highly tolerant of Cr (VI), in part, by their interaction with the endophytic chromium hyper-tolerant *Bacillus* sp. MH778713 (Ramírez et al., 2019). *Bacillus* sp. MH778713 was isolated from *Prosopis laevigata* nodules. This *Bacillus* strain tolerates up to 15000 mg/L Cr(VI), bioaccumulates up to 100 mg Cr (VI)/g of cells and promotes the development of Cr-stressed *P. laevigata* seedlings growing in hydroponic systems. Other Cr-resistant bacteria with lower tolerance (up to 780 mg/L Cr), including *Bacillus* genera, also promote the growth of Cr-stressed plants suggesting that PGPR help plants to reduce metal-induced oxidative damage (Bruno et al., 2020; Din et al., 2020; Shreya et al., 2020). Cr-tolerant bacteria with PGPR features ameliorate Cr-toxicity on plants through reduction of Cr(VI) to Cr(III) to stabilize Cr, increasing antioxidant activities of plants, reducing lipid peroxidation and removing Cr from soil (Purwanti et al., 2017; Bruno et al., 2020; Din et al., 2020). In this paper an elevated Cr(VI) concentration (2500 mg L^–1^) was required to prevent germination of imbibed seeds of *Prosopis*, highlighting the tolerance of *Prosopis* to heavy metals. Only seeds exposed to *Bacillus* sp. MH778713 volatiles were able to germinate in the presence of 2500 mg L^–1^ Cr(VI). Long-chain alkanes released by *Bacillus* sp. MH778713 were found to help seeds to overcome dormancy, the potential role of these compounds in response to chromium stress will be discussed.

## Materials and Methods

### Bacteria and Seed Preparation

*Bacillus* sp. MH778713, an isolate from *P. laevigata* nodules (Ramírez et al., 2019), and *Escherichia coli* BL21(DE3) were streaked onto yeast-extract mannitol agar (YMA) plates and incubated for 24 h in darkness at 28°C. For long-term storage, bacterial strains were kept at −80°C in yeast-extract mannitol broth with 20%(v/v) glycerol. *Prosopis laevigata* seeds were surface-sterilized by rinsing with distilled water and then soaking in concentrated sulfuric acid (98%) for 20 min, followed by rinsing 3–4 times in sterile distilled water. *Arabidopsis thaliana* ecotype Columbia (Col-0) seeds were prepared by treating them with 2% Tween-20 for 1 min, washed five times with sterile distilled water and placed on Petri dishes containing water agar (0.75% w/v). All chemicals including heneicosane, hentriacontane, 2,4-di-tert-butyl phenol, and tetracosane were purchased from Sigma Aldrich.

### Determination of Minimum Inhibitory Concentration (MIC) of Chromium to Stop Germination of *Prosopis laevigata* and *Arabidopsis thaliana*

To determine the minimal amount of Cr(VI) that stop seed germination of *Prosopis laevigata*, increasing concentrations of 100 mg L^–1^ chromium (100, 200, 300, 400 up to 2500 mg L^–1^) were assayed. For *Arabidopsis thaliana* ecotype Col-0 increasing concentrations of 10 mg L^–1^ Cr(VI) (10, 20, 30, 40 up to 100 mg L^–1^) were assayed. Higher concentrations of chromium were not assayed because *P. laevigata* and *A. thaliana* seeds did not germinate with 2500 and 100 mg L^–1^ of Cr(VI), although they did germinate with 2400 and 90 mg L^–1^, respectively. Surface-sterilized *Prosopis* or *Arabidopsis* seeds were placed onto partitioned plates containing water-agar (0.75% w/v) added with concentrations of K_2_CrO_4_ described above. Petri plates with seeds were incubated at 28°C for 24 and 72 h in dark for the germination test of *Prosopis* and *Arabidopsis*. After germination, *Prosopis* and *Arabidopsis* seedling were incubated two more days for complete radicle development. The minimum inhibitory concentration assay was done in quintuplet, each experiment was repeated at least three times.

### Effect of Volatiles Released by *Bacillus* sp. MH778713 on Germination of Cr-Stressed Seeds

**FIGURE 1:**
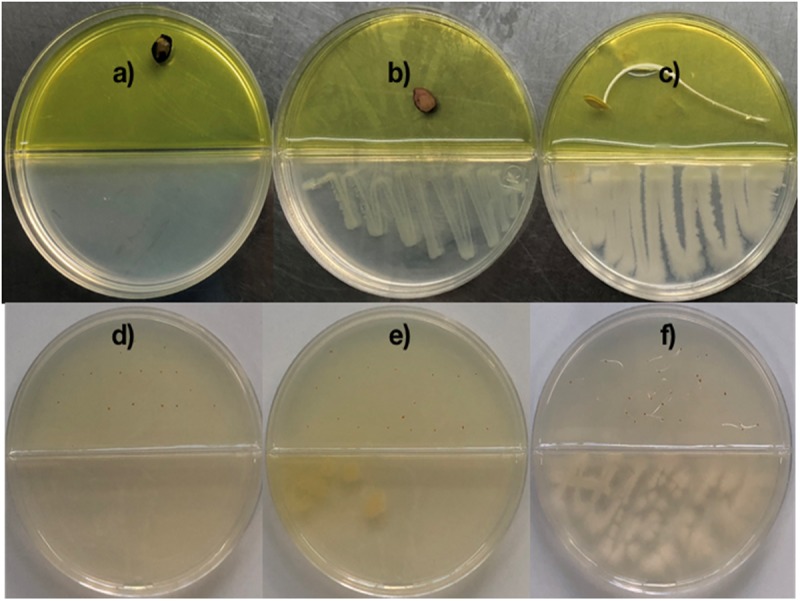
Co-cultivation of *Bacillus* sp. MH778713 and *Prosopis laevigata*/*Arabidopsis thaliana* in partitioned Petri dish in the dark at 28°C for germination assay. Pictures were taken after 72 and 120 h for *Prosopis* and *Arabidopsis*, but germination was observed after 24 and 72 h, respectively. *Prosopis laevigata* seeds were imbibed 72 h on an aqueous agar plate containing 2500 mg L^–1^ of Cr(VI) while exposed to volatiles released by *Escherichia coli* BL21 (DE3) as a negative control **(b)**, *Bacillus* sp. MH778713 **(c)** or nothing (negative control) **(a)**. Similarly, *Arabidopsis thaliana* seeds were imbibed 120 h on an aqueous agar plate containing 100 mg L^–1^ Cr(VI) while exposed to volatiles released by *E. coli* BL21 (DE3) **(e)**, *Bacillus* sp. MH778713 **(f)** or nothing **(d)**. Seed germination assays were done in quintuplet; each experiment was repeated at least three times.

To determine whether the blend of volatiles emitted by *Bacillus* sp. MH778713 could help *Prosopis or Arabidopsis* seeds to overcome Cr-stress, co-cultivation of *Bacillus* sp. MH778713 and *Prosopis/Arabidopsis* seed in a partitioned Petri dishes were carried out. Partitioned Petri dishes prevents physical contact between *Bacillus* and seeds. Thus, 10 μl of *Bacillus* sp. MH778713 (1 × 10^10^ CFU/ml) was inoculated on Yeast extract Mannitol Agar (YMA) medium on one side of the partitioned plate and *Prosopis* seed was placed on the Cr-water agar [2500 mg L^–1^ of Cr(VI), 0.75% w/v agar] medium on the other side of the plate. Similarly, *Arabidopsis* seeds were germinated on partitioned plates with Cr-water agar medium but with only 100 mg L^–1^ of Cr(VI). After inoculation, Petri dishes containing seeds were covered and sealed with parafilm to minimize volatiles leaking. Treated plates were incubated at 28°C for 24 and 72 h in the dark for seed germination test of *Prosopis* and *Arabidopsis*, respectively. Pictures of Figure 1 were taken 2 days after germination to let seedling reach a greater development. Partitioned Petri dishes with *Prosopis* or *Arabidopsis* seeds on Cr-water agar medium and YMA without *Bacillus* or with *Escherichia coli* BL21(DE3) were used as negative controls. Germination assay of Cr-stressed seeds in the presence and absence of *Bacillus* sp. MH778713 volatiles was performed in quintuplet, each being repeated at least three times.

### Analysis of Metabolites Released by *Bacillus* sp. MH778713

To determine the metabolites produced by *Bacillus* sp. MH778713, co-cultivations of *Bacillus* and *Prosopis* seeds in partitioned Petri dishes were carried out as described above but plates were incubated for 24 and 48 h. To do that, sealed Petri plates containing the *Bacillus* culture were incubated and then opened at 24 h to extract the compounds released by the bacterium while other plates were opened at 48 h. To extract the compounds released by *Bacillus* sp. MH778713, 3 ml of benzene was added into the compartment of the Petri dishes containing the *Bacillus* culture, the Petri dishes were gently shaken for 1 min and benzene was collected into microtubes. The microcentrifuge tubes were vortexed for 10 min to obtain the crude extract which was centrifuged at 13000 rpm (15865 *g*) for 5 min twice, the supernatant was collected for the gas chromatography-mass spectrometry (GC-MS) analysis. The metabolites extracted from co-cultivation of *Bacillus* sp. MH778713-*Prosopis* seed and cultures of *Bacillus* alone were analyzed by GC-MS. Similarly, *E. coli* BL21 DE3 was grown for 24 h in sealed partitioned Petri dishes with *Prosopis* seed for metabolites extraction with benzene as a negative control. GC/SM analysis was performed using Agilent 7820A Gas Chromatograph system (Santa Clara, CA, United States) with a HP-5ms column (30 m length, 0.25 mm i.d., 0.25 μm film thickness). The initial oven temperature was set at 40°C and kept for 5 min, then ramped up at a rate of 15°C/min to 240°C, and held 6.667 min, for a total run time of 25 min. The carrier gas was helium at 1.2 ml/min rate. The Agilent 5975C Gas Chromatograph/Mass Selective Detector (Santa Clara, CA, United States) was operated in the electron ionization mode at 70 eV, a source temperature of 220°C, with a continuous scan from m/z 50 to 500. The constituents of benzene extracts were identified by comparing the fragmentation patterns in mass spectra with those of the NIST08.L library and published mass spectra. To confirm the presence of 2,4-ditert-butylphenol, heneicosane, tetracosane, and hentriacontane in the benzene extracts, the retention time of the GC peaks of reference compounds run under identical conditions and mass spectra was also compared. Each sample was run in triplicate. Most abundant compounds that we identified as being emitted by *Bacillus* sp. MH778713 were tested individually for their ability to promote seed germination under Cr-stress.

### Promotion of Seed Germination and Seedling Growth of *Prosopis laevigata* and *Arabidopsis thaliana* by 2,4-Di-Tert-Butylphenol, Heneicosane, Hentriacontane, and Tetracosane Under Cr-Stress

To evaluate the ability of 2,4-Di-tert-butyl phenol (CAS# 96-76-4), heneicosane (CAS# 629-94-7), hentriacontane (CAS# 630-04-6), and tetracosane (CAS# 646-31-1) to break seed dormancy of Cr-stressed seeds; partitioned Petri dish experiments were performed as described above. A stock solution of each compound was prepared using benzene as a solvent. Two initial dosages of each compound were assayed, 50 and 500 μg, considering the concentrations reported in the literature as an optimal (0.01–100 μg) for plant-growth induction in partitioned Petri dish experiments (Fincheira and Quiroz, 2018). Thus, 6 or 60 μl of each stock solution was inoculated on Yeast extract Mannitol Agar (YMA) medium on one side of the partitioned plate and *Prosopis* or *Arabidopsis* seed was placed onto the Cr-water agar [2500 or 100 mg L^–1^ Cr(VI), 0.75% w/v agar] medium on the other side of the plate to evaluate seed germination. Partitioned Petri plates were incubated at 28°C for 24 and 72 h in the darkness for *Prosopis* and *Arabidopsis* germination; although pictures for figures were taken after 72 and 120 h, respectively. In the same way, *E. coli* BL21 (DE3) or benzene (6 μl) was inoculated on the YMA medium on one side of the partitioned plate as negative control while *Bacillus* sp. MH778713 was inoculated as a positive control. After 72 h of incubation, the growth parameters of *Prosopis* seedlings were evaluated to determine which compound was more effective (Figure 5). The promotion of seed germination was done in quintuplet; each experiment was repeated at least three times. Finally, 0.05, 0.5, and 5 μg of each compound was also evaluated for the germination assays.

### Growth Promotion of *Prosopis* Seedling by 2,4-Di-Tert-Butylphenol, Heneicosane, Hentriacontane, Tetracosane, and *Bacillus* sp. MH778713

Partitioned Petri plates were prepared as described above with YMA and water-agar (0.75% w/v) medium. No chromium was added to the seed side of the Petri dish to let seedling growth normally under this condition. On the side of the plate containing YMA medium 6 μl of solvent alone (benzene), 50 μg of 2,4-di-tert-butyl phenol, heneicosane, hentriacontane, or tetracosane was added to evaluate *Prosopis* seedling growth promotion. Furthermore, partitioned Petri plates with 10 μl of *Bacillus* sp. MH778713 (1 × 10^10^ CFU/ml) on YMA medium and *Prosopis* seeds on the water-agar medium were included as a positive control.

### Statistical Analysis

The statistical analysis of seedling growth parameters was performed using Sigma Plot (Handel Scientific Software). Data were analyzed globally by ANOVA single factor, and significant treatment effects were determined by the Tukey–Kramer *Post Hoc* test.

## Results

### Volatiles Emitted by *Bacillus* sp. MH778713 Break Seed Dormancy

Chromium is highly toxic to plants and harmful to their growth and development. To determine the minimal amount of chromium (VI) that prevent seed germination, surface-sterilized seeds of *Prosopis laevigata* and *Arabidopsis thaliana* were placed onto water-agar plates supplemented with 100, 200, 300, … up to 2500 mg L^–1^ of K_2_CrO_4_ for germination assays of *Prosopis* and 10, 20, 30, … up to 100 mg L^–1^ for *Arabidopsis*. We found out that 100 and 2500 mg L^–1^ of Cr(VI) precluded seed germination of *A. thaliana* and *P. laevigata*, confirming chromium phytotoxic effects. To assess the ability of *Bacillus* sp. MH778713 to produce volatiles and promote germination of seeds exposed to Cr(VI), co-cultivations of *Bacillus* sp. MH778713 and *Prosopis laevigata/Arabidopsis thaliana* in partitioned Petri dish were carried out over 3 and 5 days, respectively. Two types of agar-media were used in each partitioned Petri plate, YM-Agar medium on bacteria’s side and Water-Agar added with chromium on the seed side. The yellow color of the medium on the *Prosopis* seed’s side was caused by the high chromium concentration (Figures 1a–c). Partitioned Petri plates prevent any physical contact between bacteria and seeds or diffusion of non-volatile metabolites through the medium. Thus, any stimulating effect of *Bacillus* sp. MH778713 on seed germination can be attributed to the volatiles emitted by the bacterium. Our results showed that in the presence of *Bacillus* sp. MH778713, *P. laevigata* seeds on Cr-agar plates germinated in 24 h of co-cultivation and showed an efficient seedling development until 72 h (Figure 1c), while the negative control (seeds non-exposed to *Bacillus* VOCs) were unable to germinate in presence of chromium (Figure 1a). Similar results were observed with *Arabidopsis thaliana* seeds (Figures 1d,f), suggesting that *Bacillus* sp. MH778713 produces volatiles that promote germination and seedling growth under chromium phytotoxic conditions. *Escherichia coli* BL21(DE3), like any living organism, produces volatiles and was used as a second negative control (Figures 1b,e). Under chromium stress, neither *Prosopis* nor *Arabidopsis* seeds germinated when they were exposed to volatiles emitted by *E. coli* (Figures 1b,e). These results indicate that airborne signals emitted by *Bacillus* sp. MH778713 trigger seed dormancy-breaking on the partitioned Petri dish experiments. Since bacteria growing in rich media can use amino acids as a carbon source releasing ammonia, thus alkalinizing the medium, the pH of *Bacillus* and *E. coli* cultures was measured. Both bacteria were cultivated on YMA plates with an initial pH of 6.8, after 72 h the pH of *Bacillus* and *E. coli* cultures were 8 and 5.5, respectively. These results indicate that *E. coli* can be producing organic acids responsible of the pH drop.

### Long-Chain Alkanes and 2,4-Di-Tert-Butylphenol Were the Most Abundant Metabolites Produced by *Bacillus* sp. MH778713

**FIGURE 2:**
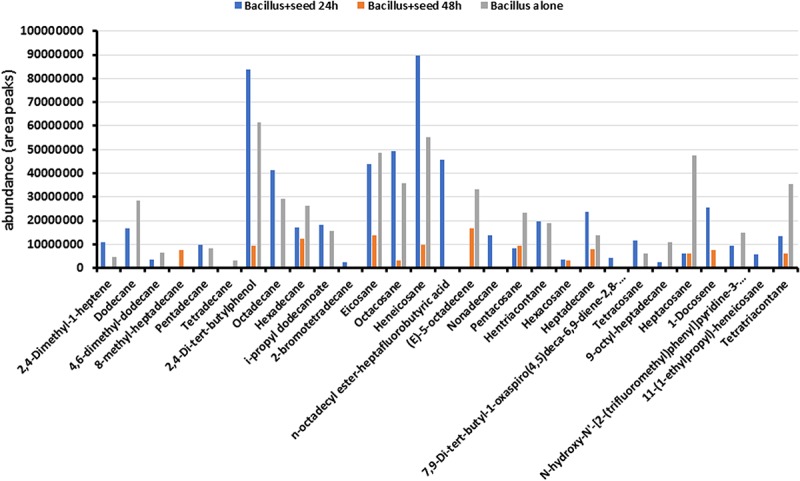
Metabolite profile of *Bacillus* sp. MH778713 produced after 24 and 48 h of co-cultivation with *Prosopis* seed in partitioned Petri dishes (blue and orange bars). Metabolite profile of *Bacillus* sp. MH778713 growing alone in partitioned Petri plate (gray bars) at 24 h of incubation.

To determine the metabolites emitted by *Bacillus* sp. MH778713, bacterial cultures like those described in Figure 1c were carried out taking samples from the Petri plates at 24 and 48 h. Benzene was used for extracting compounds produced by the *Bacillus* cultures and metabolites profile was determined using GC-MS analysis as described in section “Materials and Methods.” Nature of metabolites produced by *Bacillus* sp. MH778713 include alcohols, alkanes, alkenes, esters, ketones, amide, and fatty acids (Figure 2). A total of 29 compounds were identified, 18 of them were absent at 48 h of culture while eleven of them were present both at 24 and 48 h. The most abundant compounds produced at 24 h of culture were heneicosane, 2,4-di-tert-butylphenol, eicosane, hentriacontane, octadecane, octacosane, and *n*-octadecyl ester-heptafluorobutyric acid (Figure 2). To determine if *Prosopis* seeds were also producing volatiles during germination (24 h of incubation), the metabolite profile of *Bacillus* growing alone (without seed) in a partitioned Petri plate was determined. As shown in Figure 2, the profile of *Bacillus* with and without seed were comparable, except for 2-bromotetradecane, *n*-octadecyl ester-heptafluorobutyric acid, nonadecane, hexacosane, 7,9-Di-tert-butyl-1-oxaspiro(4,5)deca-6,9-diene-2,8-dione, 1-docosene, and 11-(1-ethylpropyl)-heneicosane that were produced only when *Prosopis* seed was present. Since culture medium and *E. coli* produce volatiles, the profile of *E. coli* growing in YMA was determined in a partitioned plate in the presence of *Prosopis* seed. No peaks related to heneicosane, 2,4-di-tert-butylphenol, eicosane, hentriacontane, octadecane, octacosane, or heptadecane were found, indicating that long-chain alkanes were certainly produced by *Bacillus* sp. MH778713. The chromatographic profiles of metabolites from *Bacillus* sp. MH778713 and *E. coli* BL21(DE3) at 24 h of incubation are shown in the Supplementary Figures S2, S3. The number of compounds produced by *Bacillus* sp. MH778713 was much higher than those produced by *E. coli*. Heneicosane, 2,4-di-tert-butylphenol, hentriacontane and tetracosane standards were used to quantify the amount produced by *Bacillus* sp. MH778713 in Petri plate, finding out that it ranged from 0.01 to 0.07 μg. The identity of heneicosane, 2,4-di-tert-butylphenol, and tetracosane produced by *Bacillus* sp. MH778713 was confirmed by comparing Kovats retention indices (KIs) with KI from pheromone data base (Supplementary Table S1). To determine if heneicosane, 2,4-di-tert-butylphenol, hentriacontane, and tetracosane were present as volatiles in the headspace of the *Bacillus* sp. MH778713 culture a partitioned plate-experiment was carried out incubating an absorbent material (zeolites) on one side of the plate and *Bacillus* culture on the other. After 24 h of incubation, volatiles from absorbent material were extracted with benzene and analyzed by GC-MS. None of four compounds was detected in the headspace of the cultures by this method used.

### Heneicosane, 2,4-Di-Tert-Butylphenol, Hentriacontane, and Tetracosane Trigger Seed Dormancy-Breaking

**FIGURE 3:**
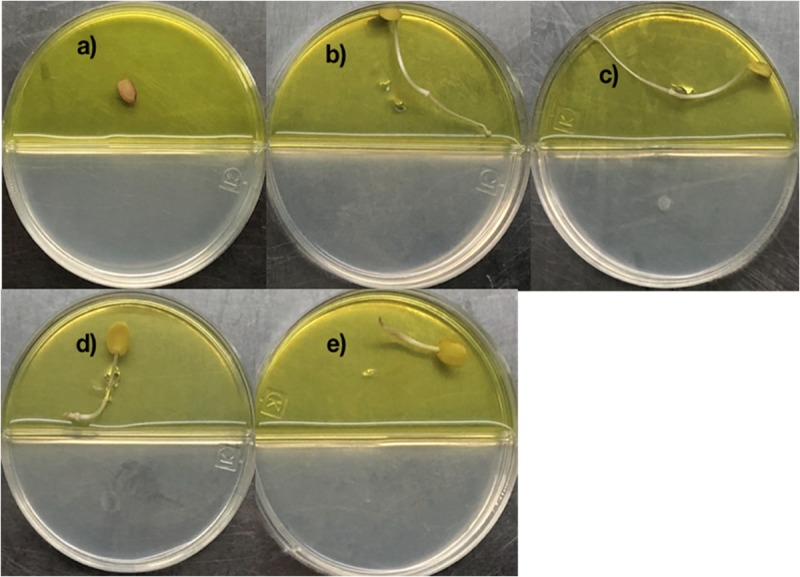
Promotion of seed germination by tetracosane, heneicosane, hentriacontane and 2,4-Di-tert-butylphenol under Cr-stress. *Prosopis laevigata* seeds were imbibed 72 h at 28°C in the dark on aqueous agar plate containing 2500 mg L^–1^ of chromium(VI) while exposed to vapor of benzene as negative control **(a)**, tetracosane **(b)**, heneicosane **(c)**, hentriacontane **(d)**, and 2,4-Di-tert-butyl phenol **(e)**. Promotion of seed germination was done in quintuplet using 50 μg of the pure compound; experiments were repeated at least three times.

**FIGURE 4:**
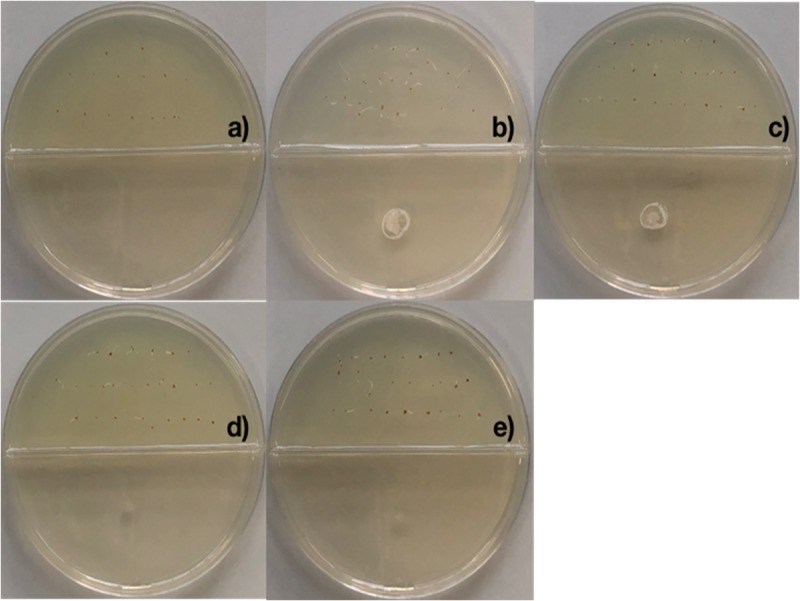
Promotion of seed germination by tetracosane, heneicosane, hentriacontane and 2,4-Di-tert-butyl phenol under Cr-stress. *Arabidopsis thaliana* seeds were imbibed 120 h at 28°C in the dark on aqueous agar plate containing 100 mg L^–1^ of chromium(VI) while exposed to vapor of benzene as negative control **(a)**, tetracosane **(b)**, hentriacontane **(c)**, heneicosane **(d)**, and 2,4-Di-tert-butyl phenol **(e)**. Promotion of seed germination was done using 25 seeds per plate, each plate added with 50 μg of the pure compound; experiments were repeated at least three times.

2,4-di-tert-butyl phenol, heneicosane, hentriacontane, and tetracosane were selected to explore their ability to induce seed dormancy-breaking. 2,4-di-tert-butylphenol was chosen because it is a well-known antioxidant and antifungal VOC produced by *Shewanella algae* YM17, *Trichoderma harzianum* T-E5, and *Arthrobacter agilis* UMCV2. Since our *Bacillus* sp. MH778713 produced alkanes range from C16 to C34 atoms, heneicosane (C_21_H_44_) was chosen as a representative compound of alkanes of 20 carbons and hentriacontane (C_31_H_64_) as a representative of alkanes of 30 carbons. Moreover, tetracosane (C_24_H_50_) was included in the assay to test if alike alkanes behave similarly (C_21_H_44_ and C_24_H_50_). Considering the VOCs concentration reported in the literature as optimal for plant-growth induction in partitioned Petri dish experiments (0.01–100 μg), we decided to test a moderate (50 μg) and high (500 μg) dosage of 2,4-di-tert-butyl phenol, heneicosane, hentriacontane, and tetracosane. All compounds induced seed germination in the presence of chromium and no difference in the stimulating effect on seed germination was observed at 50 and 500 μg dosage. The high (500 μg) dosage of 2,4-di-tert-butyl phenol, heneicosane, hentriacontane, and tetracosane did not show any phytotoxic effects on the seed germination assay of *Prosopis*. Representative pictures of seed germination experiments using 50 μg of 2,4-di-tert-butyl phenol, heneicosane, hentriacontane, and tetracosane are shown in Figure 3. For each condition tested, five *Prosopis laevigata* seeds were assayed, one per plate, over 3 days of laboratory conditions. Each seed was placed on a water-agar medium added with 2500 mg L^–1^ of chromium (VI) and 2,4-di-tert-butyl phenol, heneicosane, hentriacontane, tetracosane or benzene were inoculated on YM-agar side of the partitioned Petri dish. The negative control, benzene, was unable to induce seed germination (Figure 3a) while all assessed compounds triggered seed dormancy-breaking (Figures 3b–e). Since all stock solutions were prepared using benzene as a solvent, benzene was also tested as a growth inducer but failed to induce seed dormancy-breaking (Figure 3a). Similarly, pure compounds were tested on *A. thaliana* seeds subjected to 100 mg L^–1^ of chromium (Figure 4). Benzene failed to break seed dormancy of *A. thaliana* (Figure 4a), while 50 μg of tetracosane, hentriacontane, heneicosane, and 2,4-di-tert-butyl phenol induced 96, 44, 72, and 56% of seed germination (Figures 4b–e). Lower concentrations (0.05, 0.5, and 5 μg per plate) of each pure compound were tested for their ability to break the dormancy of *Prosopis* seeds under chromium stress. Only the dosage of 5 μg was able to break seed dormancy while 0.05 and 0.5 μg was not (Supplementary Figure S1).

**FIGURE 5:**
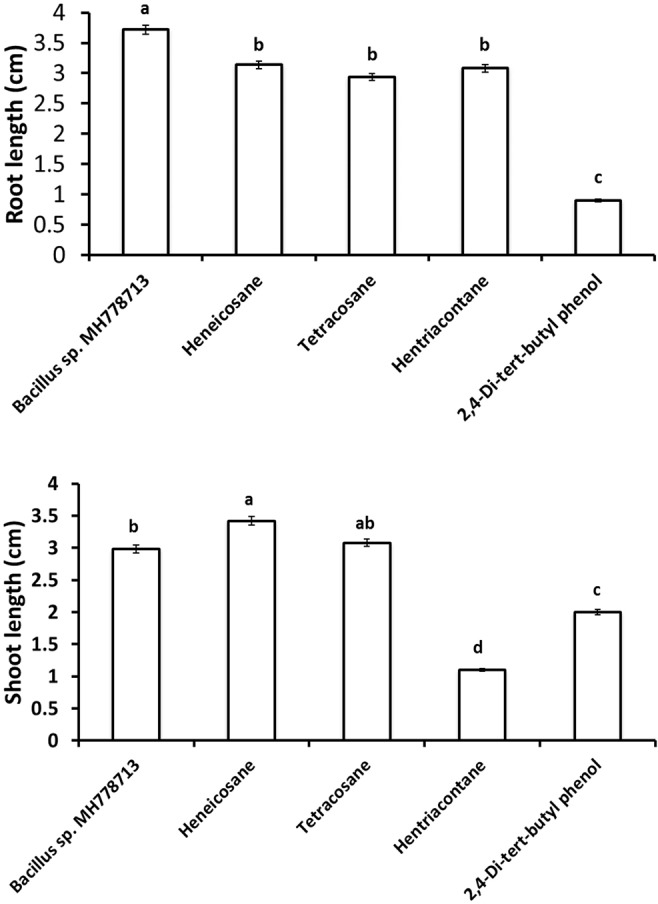
Growth promotion of *Prosopis* seedling by heneicosane, tetracosane, hentriacontane, 2,4-Di-tert-butyl phenol and volatiles released by *Bacillus* sp. MH778713 in partitioned Petri plates using 50 μg of pure compound. *Prosopis* seeds were imbibed 72 h at 28°C in the dark on an aqueous agar plate containing 2500 mg L^–1^ of chromium(VI) while exposed to volatile compounds. The promotion of seed germination was done in quintuplet; each experiment was repeated at least three times. Different letters indicate significant differences between treatments, according to the least significant difference at *P* = 0.05. Error bars represent the standard deviation of five measurements.

**FIGURE 6:**
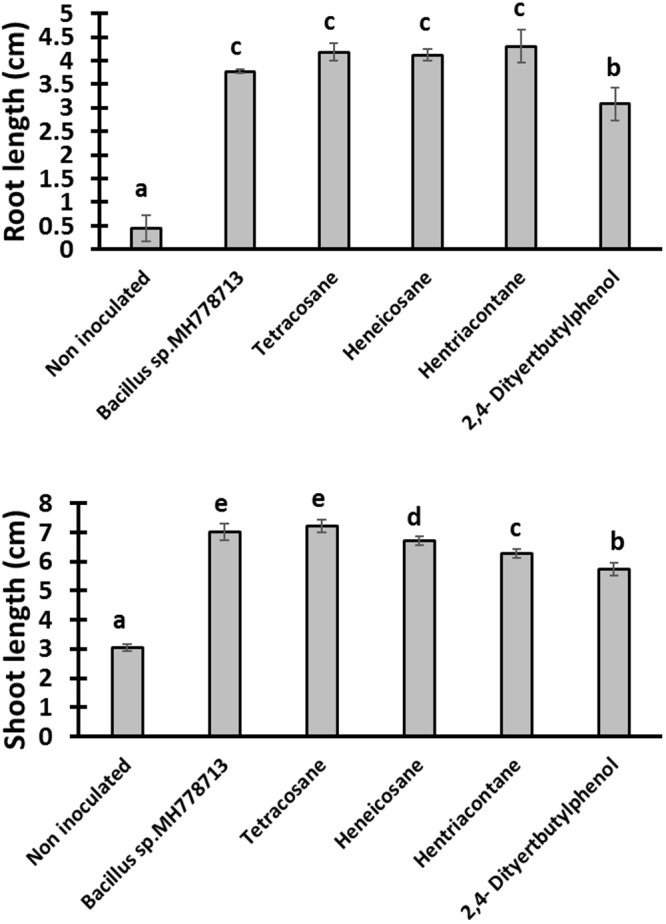
Growth promotion of *Prosopis* seedling by heneicosane, tetracosane, hentriacontane, 2,4-Di-tert-butyl phenol and volatiles released by *Bacillus* sp. MH778713 in partitioned Petri plates without chromium using 50 μg of pure compound. *Prosopis* seeds were imbibed 72 h at 28°C in the dark on aqueous agar plate while exposed to volatile compounds. The promotion of seed germination was done in quintuplet; each experiment was repeated at least three times. Different letters indicate significant differences between treatments, according to the least significant difference at *P* = 0.05. Error bars represent the standard deviation of five measurements.

The growth stimulation of *Prosopis laevigata* seedlings subjected to chromium toxicity was determined (Figure 5). The root length obtained with heneicosane (C_21_H_44_), tetracosane (C_24_H_50_) and hentriacontane (C_31_H_64_) were significantly higher than those of 2,4-di-tert-butylphenol but lower than those obtained with volatile emitted by *Bacillus* sp. MH778713. For the shoot length stimulation, heneicosane (C_21_H_44_) and tetracosane (C_24_H_50_) were as good inducers as volatiles released by *Bacillus* sp. MH778713. The growth promotion of *Prosopis* seedlings by long-chain alkanes and *Bacillus* sp. MH778713 volatiles was also evaluated without chromium stress (Figure 6). Root and shoot length of seedling exposed to *Bacillus* sp. MH778713 volatiles, tetracosane, heneicosane, hentriacontane or 2,4-di-tert-butylphenol were greater than those of the seedling control (seeds without any volatile). These results together indicate that heneicosane, 2,4-di-tert-butylphenol, hentriacontane, and tetracosane are growth inducers of *Prosopis laevigata* seedlings and allow seeds to overcome phytotoxicity imposed by chromium (VI).

## Discussion

In this work, we established the ability of *Bacillus* sp. MH778713 to produce volatiles which in turn triggered the breaking of seed dormancy under chromium stress condition. Plant growth-promoting rhizobacteria release a blend of volatile compounds such as 2,3-butanediol, 1,3-propanediol, indole, acetoin, 2-nonanone, 2-undecanone, 2-tridecanone, hexadecane, and other hydrocarbons, ketones, alcohols, terpenes, acids, and sulfurs that can stimulate plant growth, control plant pathogens, and induce systemic disease resistance (Ryu et al., 2003; Audrain et al., 2015; Fincheira et al., 2017). Different environmental signals can induce or preclude seed germination. A dormancy state can prevent imbibed viable seeds from germinating under apparently favorable conditions as an adaptive strategy (Arc et al., 2013; Nishimura et al., 2018). Heavy metals, for example, trigger signals (increasing ABA concentrations) that preclude seed germination inducing a dormancy state. Although seed coat provides protection, some metals can permeate into the seed before germination or diffuse into the embryo upon seed-coat cracking leading to alterations into nitrogen and carbohydrate metabolism, water uptake, oxidative stress, and seed germination (Kranner and Colville, 2011; Yıldız and Terzi, 2016; Kaszycki et al., 2018). Elevated Cr(VI) concentrations (75–100 mg kg^–1^ soil) inhibit seed germination, reduce chlorophyll contents and affect most of the growth parameters of biofuel plant species (Amin et al., 2019). In this work, 2500 mg L^–1^ of Cr(VI) (∼1980 mg kg^–1^ soil) was required to prevent *Prosopis laevigata* germination, highlighting the heavy metal tolerance of *Prosopis*. The high Cr concentration tested (2500 mg L^–1^) is 19-fold higher than those found in polluted soils (Amin et al., 2019); showing the bioremediation potential of using *P. laevigata* in combination with *Bacillus* sp. MH778713 for soil restoration of sites contaminated with Cr. Since the Cr-tolerant *Bacillus* sp. MH778713 is also highly tolerant to aluminum (up to 10000 mg L^–1^), it could be expected that the system *P. laevigata*-*Bacillus* sp. MH778713 could also work in aluminum polluted sites (Ramírez et al., 2019). The deleterious and signaling role of ROS in plants is well documented, thus ROS fine-tuning plays a key role in seed physiology (Bailly et al., 2008). In our experiments, elevated ROS generated by seeds subjected to Cr stress would likely prevent seed germination by repressing hormonal signaling pathways involved in the alleviation of dormancy. However, further analysis will be required to elucidate the mechanisms of Cr(VI) preventing seed germination.

Volatiles emitted by *Bacillus* sp. MH778713 allowed *P. laevigata* seeds to overcome Cr(VI) toxicity, breaking seed dormancy while developing healthy sprouts (Figure 1). Since the medium of *Bacillus* cultures was alkalinized during germination assays, probably by the release of ammonia, ammonia could probably be involved in the beneficial observed effect; nonetheless, bacterial ammonia can also cause plant growth inhibition (Weise et al., 2013). More experiments should be done to determine the contribution of ammonia in the growth of *Prosopis* seedlings. Although VOCs effect on seed subjected to chromium stress has not been studied yet, the promoting plant growth effect of VOCs through signaling networks of cytokinins, brassinosteroids, auxin, and gibberellins is well established (Ryu et al., 2003, 2005; Zhang et al., 2007, 2008; Lee et al., 2012; Park et al., 2013). Of most abundant volatiles produced by *Bacillus* sp. MH778713 at 24 h in partitioned Petri dishes, four, heneicosane, hentriacontane, tetracosane, and 2,4-di-tert-butylphenol, were tested as pure compounds and found to break the dormancy of *Prosopis* seeds subjected to Cr(VI) phytotoxicity (Figures 3, 4). Although the mechanism of long-chain bacterial compounds underlying the observed seed germination under Cr-stress remains to be elucidated, the ability of long-chain hydrocarbons to elicit plant defense against pathogens in *Arabidopsis* is already known. Tridecane and hexadecane significantly up-regulate pathogenesis-related gene 1 (PR1) for salicylic acid signaling decreasing sensitivity to biotrophic pathogen infection (Lee et al., 2012; Park et al., 2013).

*Bacillus* sp. MH778713 was found to produce long-chain hydrocarbons such as C34, C31, C28, C25, C21, and C20 alkanes that likely are VOCs influencing the seed (Figure 2). However, bacterial volatile compounds are considered odorous molecules with low molecular weight (<300 Da) and high vapor pressure (0.01 kPa at 20°C) that easily become vapors (Audrain et al., 2015). The two most abundant volatiles released by PGPR with the characteristics described above is acetoin and 2,3-butanediol. Indeed, *Bacillus subtilis* GB03 and *Bacillus amyloliquefaciens* IN937a produce more than thirty low molecular-weight bacterial volatiles (Lee et al., 2012). Nonetheless, other studies have found high molecular weight VOCs produced by PGPR. *Pseudomonas brassicacearum* and *Pseudomonas putida* for example, release long-chain hydrocarbons like triacontane (C_30_H_62_), octacosane (C_28_H_58_), heptacosane (C_27_H_56_), and heneicosane (C_21_H_44_) with molecular weight as high as 422 g/mol and vapor pressure as low as 3.6 × 10^–12^ kPa and still being detected in the headspace of agar slant tubes by solid-phase micro-extraction (SPME) method (Giorgio et al., 2015). So, finding long-chain alkanes in our results was not surprising since the quantity and identity of volatile blends produced by rhizobacteria vary significantly among species (Ryu et al., 2003; Lee et al., 2012; Farag et al., 2013; Fincheira et al., 2017; Giorgio et al., 2015). The release of long-chain alkanes (C31, C28, C27, and C21) by rhizobacteria isolated from common bean was previously reported (Giorgio et al., 2015), but the biological role of long-chain alkanes was not further assessed. Although the method used in this work failed to detect long-chain alkanes in the headspace of *Bacillus* cultures, and solid-phase micro-extraction should be used instead, the results presented herein provide evidence that *Bacillus* sp. MH778713 emits long-chain bacterial compounds (heneicosane, hentriacontane, tetracosane) that can prime signaling pathways related to abiotic stress tolerance to chromium. From the germination assays with pure compounds, it was suggested that 2,4-di-tert-butyl phenol, heneicosane, hentriacontane, and tetracosane can be volatiles but a pre-concentration system like solid-phase micro-extraction will be required to detect them in the headspace of *Bacillus* sp. MH778713 cultures (Povolo and Contarini, 2003; Zscheppank et al., 2014). Further works will be required to decipher the molecular targets within seeds sensing the metabolites signals emitted by *Bacillus* in order to elucidate a potential mechanism by which these compounds break seed dormancy.

## Conclusion

Overall, the results provide new evidence that C31, C24, C21 hydrocarbon, hentriacontane, tetracosane, and heneicosane emitted from *Bacillus* sp. MH778713 can help seeds to overcome chromium toxicity that otherwise prevent seed germination.

## Data Availability Statement

All datasets generated for this study are included in the article/Supplementary Material.

## Author Contributions

VR, J-AM, and AB conceived, designed, and directed the project, contributed to the interpretation of the results, and designed the figures. VR worked out almost all the technical details, contributed to sample preparation, carried out the experiments, and performed the numerical calculations for the suggested experiments. VR, LC, JM-R, and RP participated in the acquisition of the data. RP, JM-R, and AB supervised the work. VR, LC, and RP manufactured the samples and performed the GC-MS analysis. AB and J-AM took the lead in the writing of the manuscript. All authors provided critical feedback, helped to shape the research, analysis, and manuscript discussed the results and contributed to the final version of the manuscript.

## Conflict of Interest

The authors declare that the research was conducted in the absence of any commercial or financial relationships that could be construed as a potential conflict of interest.

## Supplementary Material

FIGURE S1 Promotion of seed germination by 5 μg of tetracosane, heneicosane, hentriacontane and 2,4-Di-tert-butyl phenol under Cr-stress. *Prosopis* laevigata seeds were imbibed 72 h at 28°C in the dark on aqueous agar plate containing 2500 mg L^−1^ of chromium(VI) while exposed to vapor of benzene as negative control **(a)**, *Bacillus* sp. MH778713 **(b)**, tetracosane **(c)**, heneicosane **(d)**, hentriacontane **(e)**, and 2,4-Di-tert-butyl phenol **(f)**. Promotion of seed germination was done in quintuplet using 5 μg of the pure compound; experiments were repeated at least three times.

FIGURE S2 Metabolite profile of *Bacillus* sp. MH778713 grown alone at 24 h of incubation in YMA sealed plate.

FIGURE S3 Metabolite profile of *Escherichia coli* BL21 (DE3) grown in co-culture with *Prosopis* seed at 24 h of incubation in YMA sealed plate.

TABLE S1 Kovats retention indices. RT, retention time; KI exp., experimental Kovats index; KI Pherobase, Kovats index from pheromone database. NA, not available.
