# Supplementary material for: Long-Chain Hydrocarbons (C21, C24, and C31) Released by Bacillus sp. MH778713 Break Dormancy of Mesquite Seeds Subjected to Chromium Stress
Source: Front Microbiol. 2020 Apr 24;11:741. doi: 10.3389/fmicb.2020.00741 (PMC7212387; doi:10.3389/fmicb.2020.00741)
Supplement: FIGURE S1 — Promotion of seed germination by 5 μg of tetracosane, heneicosane, hentriacontane and 2,4-Di-tert-butyl phenol under Cr-stress. Prosopis laevigata seeds were imbibed 72 h at 28°C in the dark on aqueous agar plate containing 2500 mg L−1 of chromium(VI) while exposed to vapor of benzene as negative control (a), Bacillus sp. MH778713 (b), tetracosane (c), heneicosane (d), hentriacontane (e), and 2,4-Di-tert-butyl phenol (f). Promotion of seed germination was done in quintuplet using 5 μg of the pure compound; experiments were repeated at least three times. [file Data_Sheet_1.PDF]

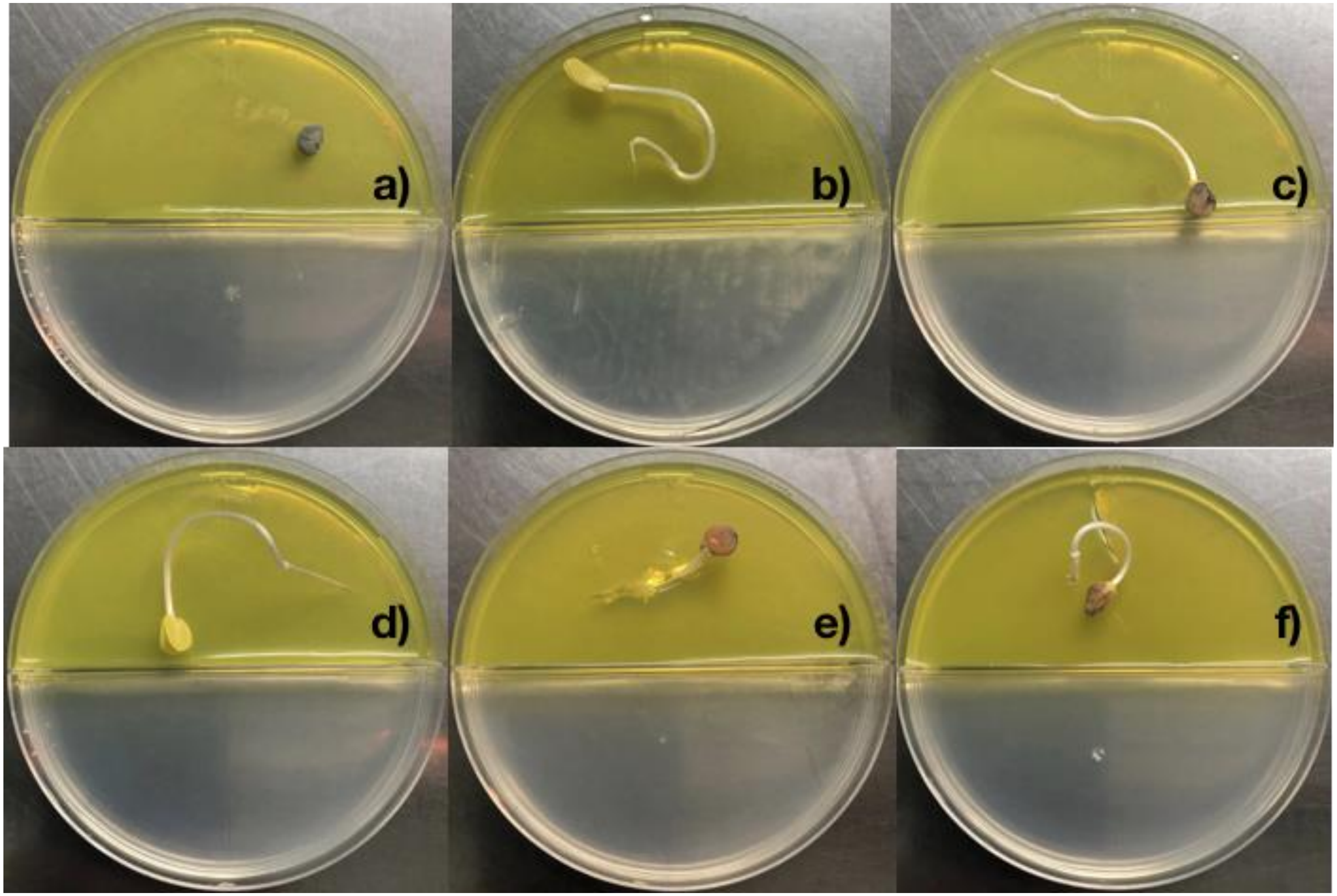

a) Benzene; b) Bacillus; c) Tetracosane; d) Heneicosane; e) Hentriacontane; f) 2,4 ditertbutylphenol. Each plate with 5 µg of pure compound.
