# Supplementary material for: Long-Chain Hydrocarbons (C21, C24, and C31) Released by Bacillus sp. MH778713 Break Dormancy of Mesquite Seeds Subjected to Chromium Stress
Source: Front Microbiol. 2020 Apr 24;11:741. doi: 10.3389/fmicb.2020.00741 (PMC7212387; doi:10.3389/fmicb.2020.00741)

Library Search Report

```
Data Path : D:\2\RPR\VERONICA 10.12.18\
Data File : BENCENO B24 02.D
Acq On    : 10 Dec 2018  19:56
Operator  : DCA
Sample    : BENCENO_B24_02
Misc      :
ALS Vial  : 3   Sample Multiplier: 1

Search Libraries:   C:\Database\NIST08.L            Minimum Quality:   0

Unknown Spectrum:   Apex
Integration Events: ChemStation Integrator - autoint1.e
```

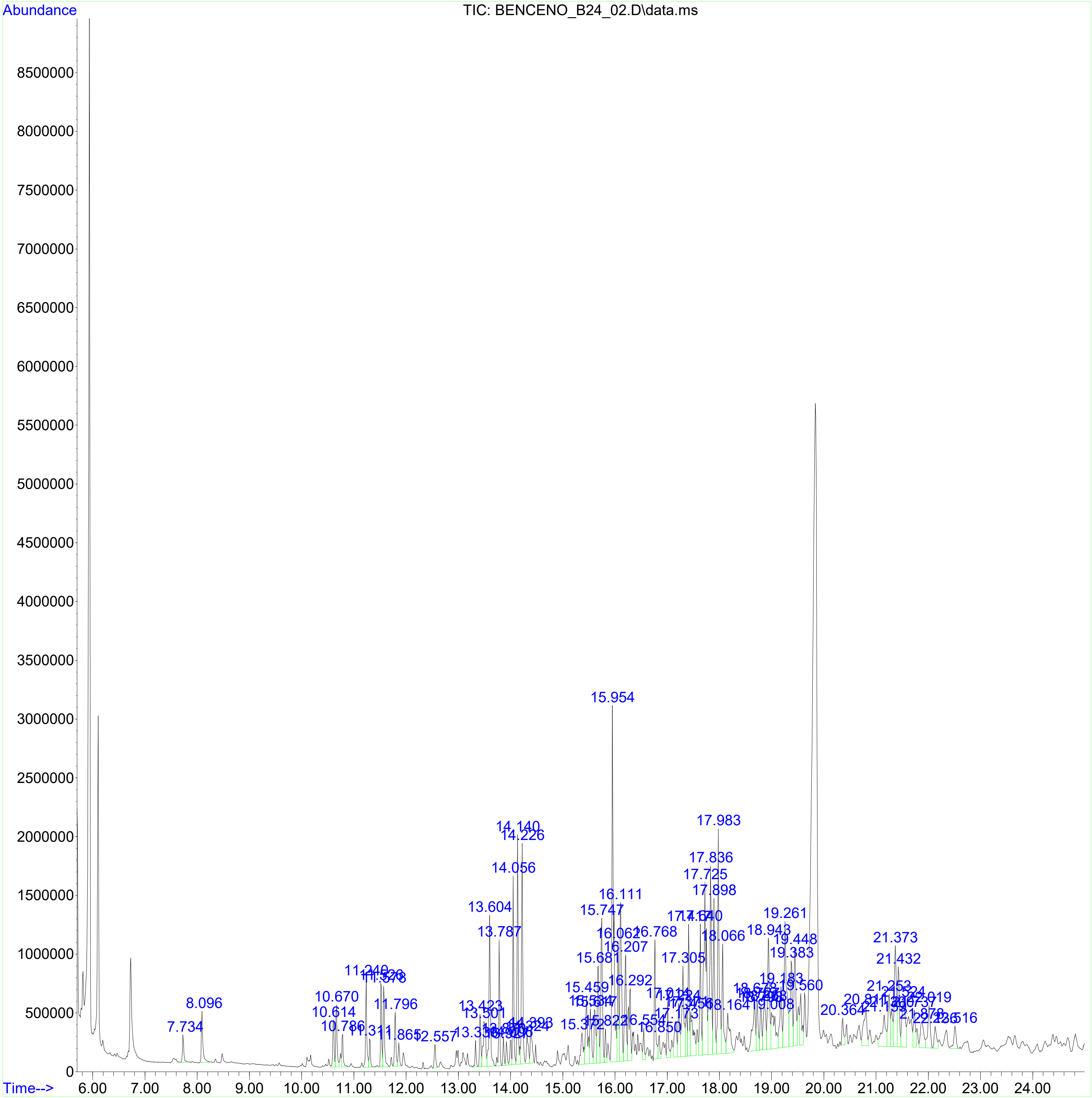

Supplement: FIGURE S2 — Metabolite profile of Bacillus sp. MH778713 grown alone at 24 h of incubation in YMA sealed plate. [file Data_Sheet_2.PDF]
