# Supplementary material for: Long-Chain Hydrocarbons (C21, C24, and C31) Released by Bacillus sp. MH778713 Break Dormancy of Mesquite Seeds Subjected to Chromium Stress
Source: Front Microbiol. 2020 Apr 24;11:741. doi: 10.3389/fmicb.2020.00741 (PMC7212387; doi:10.3389/fmicb.2020.00741)

Library Search Report

Data Path : D:\2\RPR\VERONICA 10.12.18\
Data File : BENCENO EC 02.D
Acq On   : 10 Dec 2018 18:48
Operator : DCA
Sample   : BENCENO_EC_02
Misc   :
ALS Vial : 2   Sample Multiplier: 1

Search Libraries: C:\Database\NIST08.L   Minimum Quality: 0

Unknown Spectrum: Apex
Integration Events: ChemStation Integrator - autoint1.e

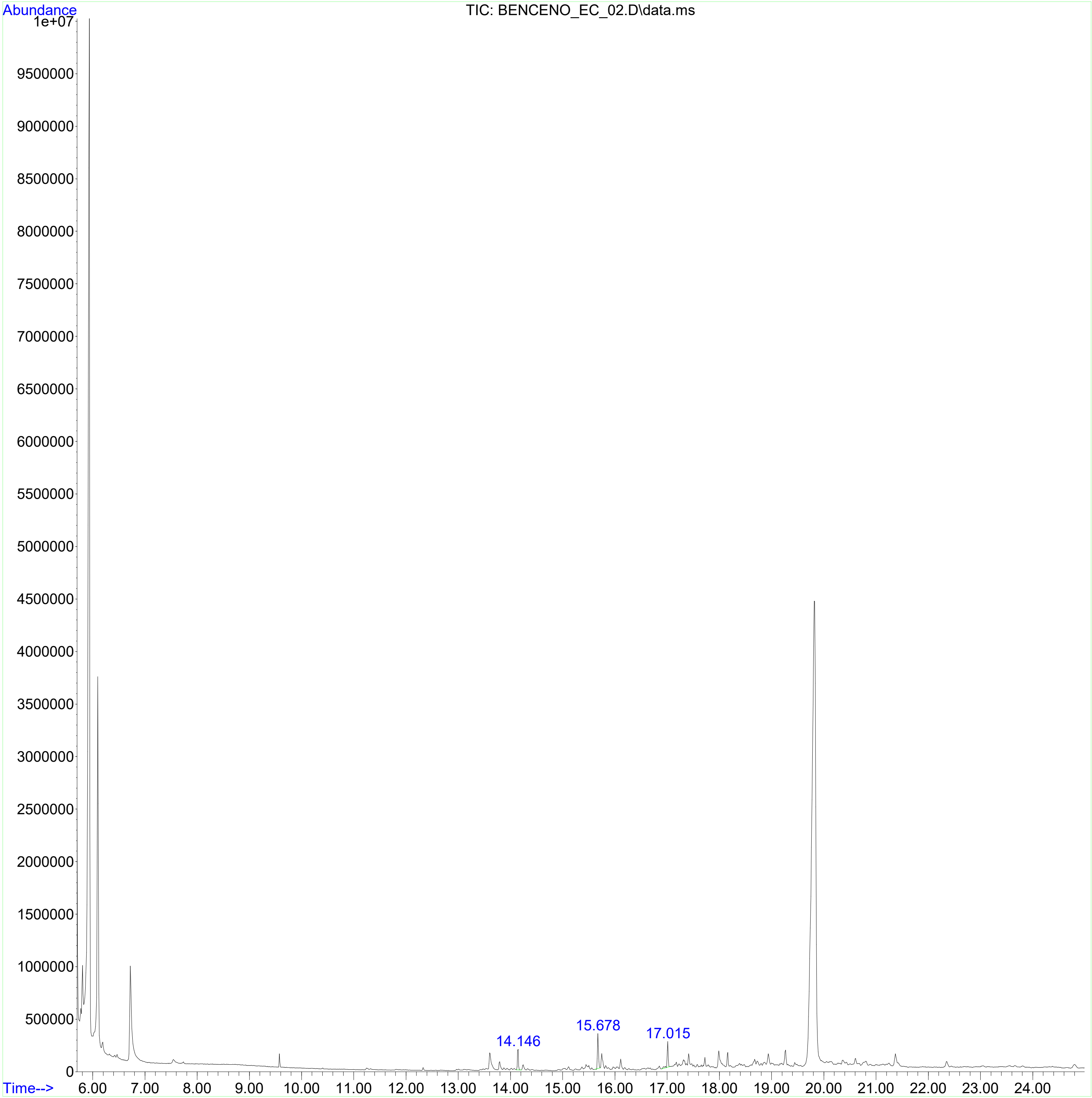

Supplement: FIGURE S3 — Metabolite profile of Escherichia coli BL21 (DE3) grown in co-culture with Prosopis seed at 24 h of incubation in YMA sealed plate. [file Data_Sheet_3.PDF]
