# Supplementary material for: Long-Chain Hydrocarbons (C21, C24, and C31) Released by Bacillus sp. MH778713 Break Dormancy of Mesquite Seeds Subjected to Chromium Stress
Source: Front Microbiol. 2020 Apr 24;11:741. doi: 10.3389/fmicb.2020.00741 (PMC7212387; doi:10.3389/fmicb.2020.00741)
Supplement: TABLE S1 — Kovats retention indices. RT, retention time; KI exp., experimental Kovats index; KI Pherobase, Kovats index from pheromone database. NA, not available. [file Table_1.DOCX]

Supplemental Table 1. Kovats retention indeces. RT= retention time, KI exp. = experimental kovats index, KI Pherobase = Kovats index from pheromone database. NA= not available.

| **Compound** | **RT (min)** | **NIST Quality (%)** | KI exp. | KI Pherobase |
| --- | --- | --- | --- | --- |
| 2,4-ditertbutylphenol | 15.95 | 96 | 1516 | 1502 |
| heneicosane | 20.362 | 97 | 2024 | 2100 |
| tetracosane | 24.463 | 96 | 2460 | 2403 |
| hentriacontane | 22.74 | 91 | NA | NA |
